# Relationships Between Brain Glucose Metabolism Patterns and Impaired Glycemic Status: A Systematic Review of FDG‐PET Studies With a Focus on Alzheimer's Disease

**DOI:** 10.1002/hbm.70180

**Published:** 2025-03-03

**Authors:** Setareh Soltani, Mahsa Dolatshahi, Sara Soltani, Kian Khazaei, Maryam Rahmani, Cyrus A. Raji

**Affiliations:** ^1^ Clinical Research Development Center, Taleghani and Imam Ali Hospital Kermanshah University of Medical Sciences Kermanshah Iran; ^2^ Mallinckrodt Institute of Radiology, Washington University in St. Louis St. Louis Missouri USA; ^3^ Sleep Disorders Research Center Kermanshah University of Medical Sciences Kermanshah Iran

**Keywords:** Alzheimer's disease, brain glucose uptake, diabetes, insulin resistance, positron emission tomography

## Abstract

It is well‐established that individuals with type 2 diabetes have an increased risk of developing cognitive impairment and Alzheimer's disease (AD). However, it is not fully determined how insulin resistance and type 2 diabetes are related to AD‐related brain glucose metabolism abnormalities. For this aim, we performed a systematic review of the studies investigating the association between cerebral glucose metabolism and glycemic status, including diabetes, insulin resistance, or hyperglycemia. Medline, Embase, and Cochrane databases were searched (till February 2, 2025). All English full‐text papers studying 18F‐FDG‐PET that investigated the association between cerebral FDG uptake or cerebral metabolism rate and glycemic status were included. These studies were reviewed for quality assessment, data extraction, and qualitative synthesis. After screening titles and abstracts of 718 unique records identified from our search, 23 studies (5308 participants) addressing the association between brain glucose metabolism alterations, as assessed by FDG‐PET scan, and glycemic status were included for qualitative analysis. Of these 23 studies, 22 studies suggested that hyperglycemia or insulin resistance is related to global or regional cerebral glucose hypometabolism. The regional brain metabolism reductions were mostly in the frontal cortex, parietotemporal cortex, posterior cingulate cortex, and precuneus cortex, known as AD‐signature areas. Hyperglycemia, diabetes, and insulin resistance are associated with cerebral glucose hypometabolism in similar regions compared to AD. This can suggest that even in cognitively normal individuals, insulin resistance can potentially increase the predisposition to abnormal AD‐like glucose metabolism.

## Graphical abstract

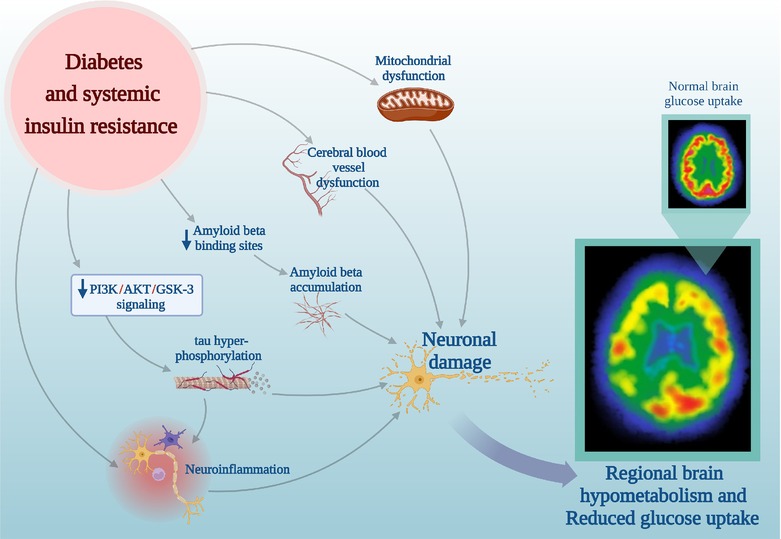

Insulin resistance can lead to neuronal damage and cerebral hypometabolism through different mechanisms including mitochondrial dysfunction, impairment of cerebral blood vessel function. In addition, insulin resistance is linked to amyloid and tau accumulation and neuroinflammation, the key mechanisms involved in Alzheimer disease and neuronal damage, leading to hypometabolism in Alzheimer disease specific regions.

**Summary**

- Question
  ○ Do the patterns of brain FDG‐PET uptake or cerebral glucose hypometabolism in individuals with type 2 diabetes or insulin resistance resemble glucose metabolism patterns in Alzheimer's disease (AD)?
- Pertinent findings
  ○ Based on most of the studies, diabetes, and insulin resistance are associated with lower brain FDG PET uptake and cerebral glucose hypometabolism, especially in AD‐related regions, including parietotemporal, posterior cingulate, and precuneus cortices, even in cognitively unimpaired individuals.
- Implications for patient care
  ○ These findings reinforce that insulin resistance can contribute to glucose hypometabolism and the risk of AD.
  ○ Also, the importance of considering glucose levels in standard FDG‐PET acquisition is further emphasized.

## 1 Introduction

Alzheimer's disease (AD) is the most common type of dementia and the most common neurodegenerative disease and is currently the sixth cause of death in the United States (Querfurth and LaFerla 2010). It is estimated that the prevalence of AD will triple worldwide by 2050, along with the aging of the population, becoming a major contributor to mortality and morbidity (Liang et al. 2021; Scheltens et al. 2021). Thus, it is imperative to recognize and modify the risk factors contributing to its development. Generally, AD is characterized by amyloid‐beta peptide (Aβ) accumulation, impaired brain metabolism, altered neurotransmission, and neuronal loss (Querfurth and LaFerla 2010), and it is important to recognize how AD risk factors contribute to each of these characteristics.

Insulin resistance (IR), defined as the inability of insulin to increase the uptake of glucose by cells, is associated with obesity, type 2 diabetes mellitus (T2DM) and pre‐diabetes (Ishibashi et al. 2014; Kullmann et al. 2020; Hill et al. 2021). A total of 463 million people (9.3% of the global adult population) were estimated to be living with diabetes in 2019, with the number expected to increase to 578 million (10.2%) in 2030 (Saeedi et al. 2019). Studies have shown that individuals with T2DM have an increased risk of developing cognitive impairment and AD, probably through cerebral IR (Stingl et al. 2012; Kullmann et al. 2016, 2020; Hölscher 2020). However, the exact pathophysiological mechanisms underlying the link between T2DM and AD are not well understood, and multiple pathways in diabetes that may lead to neuronal damage and cognitive decline have been proposed (Strachan et al. 2011; García‐Casares et al. 2014). T2DM and IR are associated with smaller total brain volume and progressive atrophy, as well as higher Aβ deposition in regions affected by early AD (Willette et al. 2013; Willette, Johnson, et al. 2015; Zhang et al. 2022). It is also suggested that IR leads to downregulated Aβ oligomer binding sites in the synapse, which causes tau protein hyperphosphorylation and accumulation of Aβ, respectively, and further results in synaptic and neuron loss (Smolina et al. 2015; Yang et al. 2018).

Currently, the most recognized neuroimaging method for estimating cerebral glucose metabolism is the 18‐fluorodeoxyglucose positron emission tomography (FDG‐PET), which also provides information on neuronal function and synaptic density (Ishibashi, Wagatsuma, et al. 2016). Generally, during the early stages of AD, FDG‐PET reveals a pattern of regional hypometabolism in the posterior cingulate cortex, and with disease progression, it expands to temporoparietal regions (Mosconi et al. 2007; Mosconi and McHugh 2011).

Studies have demonstrated that diabetes and chronic IR, even among cognitively normal individuals, lead to reduced cerebral FDG uptake in AD‐related regions (Heurling et al. 2020). Looking into the relation between cerebral glucose metabolism and IR in cognitively normal individuals and how it corresponds to AD‐related glucose metabolism patterns would provide insight into whether peripheral IR can potentially contribute to AD‐related glucose metabolism alterations. In addition, since the development of diabetes‐related cognitive impairment is a chronic process occurring in the preclinical phases of AD, the early detection of any potential alterations in cerebral metabolism induced by diabetes and IR would be helpful in designing preventive measures for AD (Chen et al. 2021).

To this aim, in this article, we reviewed the studies using FDG‐PET scan to assess the global and regional cerebral glucose metabolism (rCMRglc) and standardized uptake value ratio (SUVR) of glucose associated with impaired glycemic status, including hyperglycemia, IR, and diabetes mellitus.

## 2 Methods

### 2.1 Search Strategy and Study Selection

This systematic review and meta‐analysis study was prepared in accordance with guidelines recommended in the preferred reporting items for systematic reviews and meta‐analyses (PRISMA) checklist (Liberati et al. 2009).

Studies were identified through an electronic search of MEDLINE (PubMed), Scopus, Embase, and Cochrane library databases. No limitations for language or year of publications were applied. The search was last updated February 2, 2025. Furthermore, the reference lists of included articles and any relevant reviews were checked to ensure that all relevant studies were captured. We used the following search terms: (“Fluorodeoxyglucose F18” OR “Positron‐Emission Tomography” OR “FDG‐PET” OR “18F‐FDG PET” OR “18F‐FDG” OR “FDG” OR “18F fluorodeoxyglucose” OR “Cerebral Glucose Uptake” OR “Cerebral Glucose Metabolism” OR “Brain Glucose Metabolism” OR “Cerebral Hypometabolism”) AND (“Diabetes Mellitus” OR “Insulin Resistance” OR “Diabetes” OR “Insulin Resistance” OR “Hyperglycemia” OR “Blood Glucose” OR “Plasma Glucose level” OR “Glucose Loading” OR “Serum Glucose Level” OR “Serum Glucose Levels”) AND (“Alzheimer Disease” OR “Dementia” OR “Alzheimer's disease” OR “Cognitive Impairment” OR “Cognitively” OR “Cognition” OR “Alzheimer Disease” OR “Dementia”).

18F‐FDG‐PET studies that reported the association of cerebral uptake or metabolism with glycemic status (i.e., diabetes, IR, or hyperglycemia) were included. Studies were excluded when there was any comorbidity that could interfere with the relationship between glycemic status and cerebral uptake or metabolism, including patients who had an active tumor, prior chemotherapy, or any neurologic/psychiatric conditions other than cognitive impairment that would compromise the interpretation of data. Duplicate reports were also excluded. For eligibility assessment, two independent reviewers screened titles and abstracts and then full texts. Disagreements between the reviewers were resolved by consensus or a third reviewer.

### 2.2 Data Collection

Three reviewers independently extracted data from included studies using a data extraction sheet, and the fourth reviewer reassessed the extracted data. Disagreements between the three reviewers were resolved through discussion, and if no agreement could be reached, the decision was made by a fourth senior investigator. The following data were extracted from each study: first author's name, year of publication, study design, number of patients, study groups, and duration of study. For each individual, the following data were recorded: sex, age, body mass index (BMI), education, glycemic status (i.e., diabetes mellitus, prediabetic, or nondiabetic), blood sugar levels at the time of FDG‐PET scan, HbA1C level, homeostasis model assessment of IR (HOMA‐IR), cognitive status or mean cognitive score, family history of AD, and APOE‐ε4 allele status. The methods for image acquisition and analysis and the results of the study in terms of the association between glucose levels, IR, or diabetic status and brain glucose metabolism were also extracted.

### 2.3 Risk of Bias

Two reviewers assessed the quality of all selected articles using the Newcastle–Ottawa scale (NOS) checklist for observational studies (Ga 2000; Ma et al. 2020). If there were any disagreements between the raters, a third reviewer made the decision. The NOS was developed to assess the quality of nonrandomized studies with a “star system” in which a study is qualified on three broad perspectives: the selection of the study exposure/case and control groups, the comparability of the exposure/case and control groups; and the ascertainment of either the exposure/outcome of interest. Based on the number of stars given to each study, up to three stars mean very high risk, four to six stars mean high risk, and seven to nine stars are judged as a high‐quality study.

## 3 Results

**FIGURE 1:**
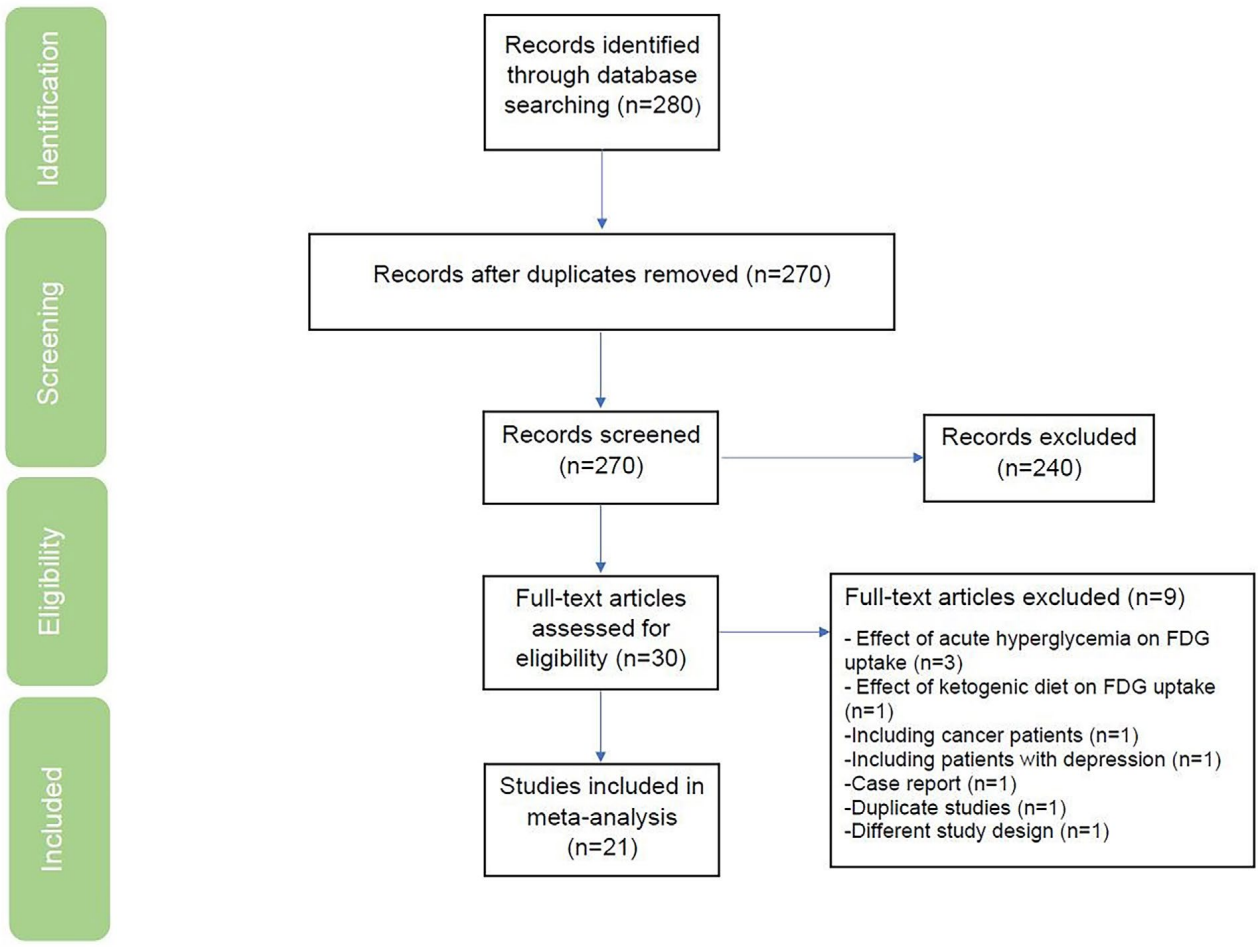
PRISMA flow diagram for study selection process. PubMed, Embase, and Cochrane were searched from inception to December 20th, 2022.

A total of 718 unique records were identified from our search. After screening titles and abstracts for potential relevance, 32 full‐text articles were retrieved and underwent full review according to the inclusion and exclusion criteria. Nine articles were excluded for various reasons (Figure 1): three studies assessed the effect of acute hyperglycemia induction on FDG uptake (Kawasaki et al. 2008; Ishibashi, Kawasaki, et al. 2015; Ishibashi, Wagatsuma, et al. 2016), one study evaluated the effect of the ketogenic diet on FDG uptake (Bennett et al. 2022), one study included patients with depression (Marano et al. 2014), one study was a case report (Ishibashi et al. 2014), one study included cancer patients who had undergone a PET scan for cancer staging (Sarikaya et al. 2020), one study was a duplicate (Garcia‐Casares et al. 2014), and one study had a different design, inducing hyperglycemia by intravenous infusion of somatostatin and insulin (Hasselbalch et al. 2001). In total, 23 studies addressed the association between brain glucose metabolism alterations, assessed by FDG‐PET scan, and cognitive impairment in glycemic impairment and were included for qualitative analysis (Figure 1).

### 3.1 Study Characteristics

Characteristics of the 23 included studies are summarized in Supporting Information S1: Tables 1 and 2. Publication dates ranged from 2011 to 2024. Among these, 11 cross‐sectional, 6 case–control, 4 cohort, and 1 case series study with a sum of about 5308 participants, 917 of whom had abnormal glycemic status, that is, diabetes or prediabetes, were included in this systematic review. Of the 23 included studies, 14 consisted of only cognitively normal individuals (a total of 1811 participants), while 9 studies included both cognitively normal individuals and patients with cognitive impairment, including MCI and AD (a total of 1861 MCI and 276 AD patients). Of approximately 5308 participants, 1967 had the APOE‐ε4 allele, and 307 had a positive AD family history, respectively.

In total, 12 studies were conducted in the United States, three in Japan, and eight in other countries (including Germany, China, Spain, Canada, France, Hungary, Australia, and the Netherlands). Five studies utilized AD Neuroimaging Initiative data for their analysis.

All included studies applied quantitative evaluations of the PET scans. A total of 18 studies used the SUVR, 2 of which reported using pons as the reference region, 1 used whole‐brain, pons, or cerebellum, and another 1 used the visual cortex, but the others did not report the reference region. Three studies reported the use of cerebral metabolic rate of glucose (CMRglu) as the outcome measure.

Seven of the 23 studies performed PET analysis based on the combination of voxel‐wise and region of interest (ROI) analysis on attenuation‐corrected images. Eight studies performed only ROI analysis, while eight studies performed only voxel‐wise analysis.

### 3.2 Quality Assessment

The mean study quality score for the included studies was 7.78 (±Standard deviation [SD]: 0.88, range: 6–9), which suggested good quality overall. Two studies had a high risk of bias and the other 21 studies had high quality (Tables [Link], [Link]). Meta‐analysis was not performed, as the study samples were heterogeneous, and there was no single statistical summary measurement (e.g., effect size) to quantify the impact of IR or blood glucose levels on SUVRs or rCMRglu.

### 3.3 Study Outcomes

Of the 23 included studies, 22 studies reported that impaired glycemic status is associated with lower cerebral FDG uptake. Among these 22 studies, a total of 14 studies reported lower rCMRglc only in specific regions, 6 studies reported both global and regional cerebral hypometabolism, while 2 studies only mentioned lower global cerebral metabolism.

A total of 20 studies (with a mixed population consisting of both cognitively normal and patients with cognitive impairment) showed that impaired glycemic status is associated with reduced cerebral FDG uptake and glucose metabolism in cognitively normal individuals. Two studies, including one cohort and one cross‐sectional study, observed this negative relationship between higher IR and lower cerebral FDG uptake only in AD and MCI patients, respectively (Willette, Modanlo et al. 2015; Li et al. 2016). Of the 20 studies reporting regional brain hypometabolism in association with IR or hyperglycemia in cognitively normal individuals, 12 studies found it in the frontal cortex (Baker et al. 2011; Burns et al. 2013, 2018; Garcia‐Casares et al. 2014; Rasgon et al. 2014; Castellano et al. 2015; Willette, Bendlin, et al. 2015; Ishibashi, Onishi, et al. 2016; Viglianti et al. 2019; Chen et al. 2021; Képes et al. 2021; Karayiannis et al. 2024), 12 studies in the parietal cortex (Baker et al. 2011; Burns et al. 2013, 2018; Roberts et al. 2014; Castellano et al. 2015; Willette, Bendlin, et al. 2015; Ishibashi et al. 2017; Viglianti et al. 2019; Sundermann et al. 2021; Palix et al. 2022; Karayiannis et al. 2024), 12 studies in the temporal cortex (Baker et al. 2011; Burns et al. 2013; Garcia‐Casares et al. 2014; Roberts et al. 2014; Castellano et al. 2015; Willette, Bendlin, et al. 2015; Ishibashi et al. 2017; Viglianti et al. 2019; Chen et al. 2021; Sundermann et al. 2021; Palix et al. 2022; Karayiannis et al. 2024), eight studies in the precuneus cortex (Burns et al. 2013, 2018; Ishibashi, Onishi, et al. 2015; Ishibashi, Onishi, et al. 2016; Ishibashi et al. 2017; Apostolova et al. 2018; Képes et al. 2021; Palix et al. 2022), and seven studies in the posterior cingulate cortex (Baker et al. 2011; Burns et al. 2013, 2018; Roberts et al. 2014; Ishibashi, Onishi, et al. 2016; Sundermann et al. 2021; Palix et al. 2022). Also, six studies reported an rCMRglc decline in the occipital cortex (Burns et al. 2013; Apostolova et al. 2018; Chen et al. 2021; Képes et al. 2021; Palix et al. 2022; Karayiannis et al. 2024), two studies in the cerebellum (Garcia‐Casares et al. 2014; Chen et al. 2021), and one study in the medial temporal lobe (Willette, Bendlin, et al. 2015).

Of the 23 studies, 10 studies assessed the relationship between cerebral FDG uptake and blood glucose levels, while 7 studies assessed the association between FDG uptake and IR as measured by HOMA‐IR. In addition, six studies compared FDG uptake in diabetes with controls.

Despite the abovementioned results on the association between impaired glycemic status and reduced cerebral FDG uptake, one of the studies by Ennis et al. (2021) showed conflicting results, that is, no association between HOMA‐IR levels and cerebral FDG uptake in cognitively normal individuals.

#### 3.3.1 AD‐Related Regions vs. AD‐Nonrelated Regions

Hypometabolism in the temporoparietal, posterior cingulate, and precuneus cortices is characteristic of the early stages of AD on FDG‐PET (Tripathi and Murray 2022). In total, 11 studies with a combined total of 1581 cognitively normal participants reported cerebral hypometabolism in at least one of the AD‐related regions, including the parietotemporal cortex, posterior cingulate, and precuneus (Burns et al. 2013, 2018; Roberts et al. 2014; Castellano et al. 2015; Ishibashi, Onishi, et al. 2015; Ishibashi, Onishi, et al. 2016; Ishibashi et al. 2017; Willette, Bendlin, et al. 2015; Chen et al. 2021; Képes et al. 2021; Karayiannis et al. 2024). Among these 10 studies, 2 studies, including 204 participants (one cohort and one cross‐sectional study), reported cerebral hypometabolism in all of the mentioned AD‐related regions (Burns et al. 2013, 2018). One study observed changes in all except the posterior cingulate (Ishibashi et al. 2017) and one study spared the temporal cortex (Ishibashi, Onishi, et al. 2016). In addition, there were two reporting a decline in cerebral glucose metabolism only in the precuneus cortex (Ishibashi, Onishi, et al. 2015; Képes et al. 2021) and two studies in the parietotemporal cortex (Castellano et al. 2015; Chen et al. 2021).

In advanced AD, cortical hypometabolism in FDG‐PET typically extends into the frontal lobes, especially the prefrontal association cortex (Brown et al. 2014; Tripathi and Murray 2022). Among the 14 studies consisting of only cognitively normal participants, nine studies reported cerebral hypometabolism in association with hyperglycemia or IR in the frontal cortex (Burns et al. 2013, 2018; Rasgon et al. 2014; Castellano et al. 2015; Willette, Bendlin, et al. 2015; Ishibashi, Onishi, et al. 2016; Chen et al. 2021; Képes et al. 2021; Karayiannis et al. 2024). Three studies also indicated hypometabolism in the prefrontal cortex (Burns et al. 2013, 2018; Rasgon et al. 2014). In addition, two studies reported impaired glycemic status associated with cerebral hypometabolism in the frontal cortex of AD and MCI patients, respectively (Willette, Modanlo et al. 2015; Li et al. 2016).

The typical AD pattern of cerebral hypometabolism in FDG‐PET shows sparing of the occipital cortex, sensorimotor cortex, cerebellum, basal ganglia, and thalamus (Hoffman et al. 2000; Mosconi et al. 2008). Among the 14 studies consisting of only cognitively normal participants included in this review, only 4 reported impaired glycemic status‐associated cerebral hypometabolism in the occipital cortex (Burns et al. 2013; Apostolova et al. 2018; Chen et al. 2021; Képes et al. 2021; Palix et al. 2022). In addition, there was one study reporting hypometabolism in the cerebellum (Chen et al. 2021).

#### 3.3.2 Hyperglycemia and Cerebral Glucose Metabolism

Of the 23 studies, 10 studies, including 2193 participants, assessed the relationship between cerebral FDG uptake and blood glucose levels. All of these 10 studies, including 6 cross‐sectional, 2 cohorts, 1 case–control, and 1 case series, indicated a negative relationship between blood glucose levels and cerebral metabolism (Burns et al. 2013, 2018; Ishibashi, Onishi, et al. 2015; Ishibashi, Onishi, et al. 2016; Ishibashi et al. 2017; Apostolova et al. 2018; Viglianti et al. 2019; Sundermann et al. 2021; Palix et al. 2022; Rajendrakumar et al. 2024). Of the 10 studies reporting the negative relationship between cerebral FDG uptake and blood glucose levels, 5 consisted only of cognitively normal participants (Burns et al. 2013, 2018; Ishibashi, Onishi, et al. 2015; Ishibashi, Onishi, et al. 2016; Ishibashi et al. 2017).

In a cohort study by Burns et al. (2018), longitudinal increases in blood glucose levels of 80 cognitively normal nondiabetic participants over 4 years of follow‐up were associated with longitudinal regional cerebral 18F‐FDG uptake decline in AD‐related regions. In another cohort study consisting of 911 participants, impaired fasting blood sugar (FBS = 100–125) at baseline was associated with lower regional cerebral 18F‐FDG uptake in AD‐related regions and higher cognitive decline 4 years later, especially in women (Sundermann et al. 2021).

#### 3.3.3 IR and Cerebral Metabolism

In addition, seven studies, including 777 participants in two cohorts, three cross‐sections, and two case controls, assessed the association between FDG uptake and IR as measured by HOMA‐IR (Baker et al. 2011; Rasgon et al. 2014; Castellano et al. 2015; Willette, Bendlin, et al. 2015; Willette, Modanlo et al. 2015; Chen et al. 2021; Ennis et al. 2021). Six studies, including 708 participants, four of which consisted only of cognitively normal participants, indicated a negative relationship between HOMA‐IR levels and cerebral metabolism (Baker et al. 2011; Rasgon et al. 2014; Castellano et al. 2015; Willette, Bendlin, et al. 2015; Willette, Modanlo et al. 2015; Chen et al. 2021). However, in the study by Ennis et al. (2021) consisting of 69 cognitively normal participants, there was no association between HOMA‐IR levels and cerebral FDG uptake.

#### 3.3.4 Diabetes and Cerebral Metabolism

Of about 5308 participants, 475 and 442 were diabetic and prediabetic, respectively. Six studies, including three case control and three cross‐sectional studies, consisting of a total of 2338 participants, of whom 398 were diabetic, reported that cerebral FDG uptake is lower in diabetes (Garcia‐Casares et al. 2014; Roberts et al. 2014; Li et al. 2016; Waqas et al. 2019; Képes et al. 2021; Karayiannis et al. 2024). Out of six studies reporting the negative relationship between cerebral FDG uptake and diabetes, four consisted only of cognitively normal participants (Roberts et al. 2014; Waqas et al. 2019; Képes et al. 2021; Karayiannis et al. 2024). However, in a cross‐sectional study including 1278 participants, such an association between diabetes and FDG uptake was reported only in MCI patients (Li et al. 2016).

## 4 Discussion

Our systematic review highlights the association of impaired glycemic status and IR with reduced regional cerebral glucose uptake, especially in AD‐related regions, including the parietotemporal, posterior cingulate, and precuneus cortices. In summary, most of the literature showed a negative relationship between blood glucose/IR levels and rCMRglc in at least one of the early AD‐related regions and in the frontal cortex.

Studies have shown that individuals with T2DM have a 1.5‐ to 2.5‐fold greater risk of developing dementia and AD (Willette et al. 2013; Ninomiya 2019). A recent meta‐analysis of brain magnetic resonance imaging (MRI) studies suggested that individuals with T2DM had significantly smaller total brain volume, including both gray matter and white matter, and approximately 1%–4% smaller hippocampal volume (Zhang et al. 2022). Higher IR is also related to progressive atrophy in the prefrontal cortex, precuneus cortex, medial temporal lobe, and parietal cortex, the regions affected by early AD (Willette et al. 2013). Moreover, higher IR corresponded to higher Aβ deposition in frontal and temporal areas, as indicated by Pittsburgh Compound B (PiB)‐PET scan (Willette, Johnson, et al. 2015) as well as elevated cerebrospinal fluid (CSF) phosphorylated tau181 (P‐tau181) and CSF total tau (T‐tau) (Laws et al. 2017; Westwood et al. 2017). In addition, an FDG‐PET study of a 70‐year‐old prediabetic man with MCI during the hyperglycemic state (BS: 162 mg/dL) showed lower uptake in the left lateral occipital area and precuneus/cuneus area, consistent with the AD‐like pattern, without any association with amyloid accumulation and AD pathology (Ishibashi et al. 2014).

In addition to the potential effects of impaired glycemic status on imaging markers of AD, hyperglycemia influences the interpretation of FDG‐PET data. For instance, a cross‐sectional study on a cancer population by Sarikaya et al. (2020) suggested that hyperglycemia (BS: 171–200 mg/dL) induced a 60.7% reduction in the whole brain 18F‐FDG uptake compared with the normoglycemic group, while hypoglycemia did not seem to affect cerebral FDG uptake. As FDG competes with glucose for transport into the brain and hexokinase phosphorylation, it can be understood that global cerebral FDG uptake is affected by plasma glucose levels (Heurling et al. 2020). However, as the relationship between blood glucose levels and brain FDG uptake is demonstrated to be nonlinear, it might not be explained only by a competitive inhibition mechanism (Viglianti et al. 2017; Sprinz et al. 2018). Animal studies suggested that in the hypoglycemic to the euglycemic range, brain glucose uptake behaves in a transporter‐limited mode but turns into an intracellular phosphorylation‐limited mode in hyperglycemia (Crane et al. 1983; Viglianti et al. 2017).

**FIGURE 2:**
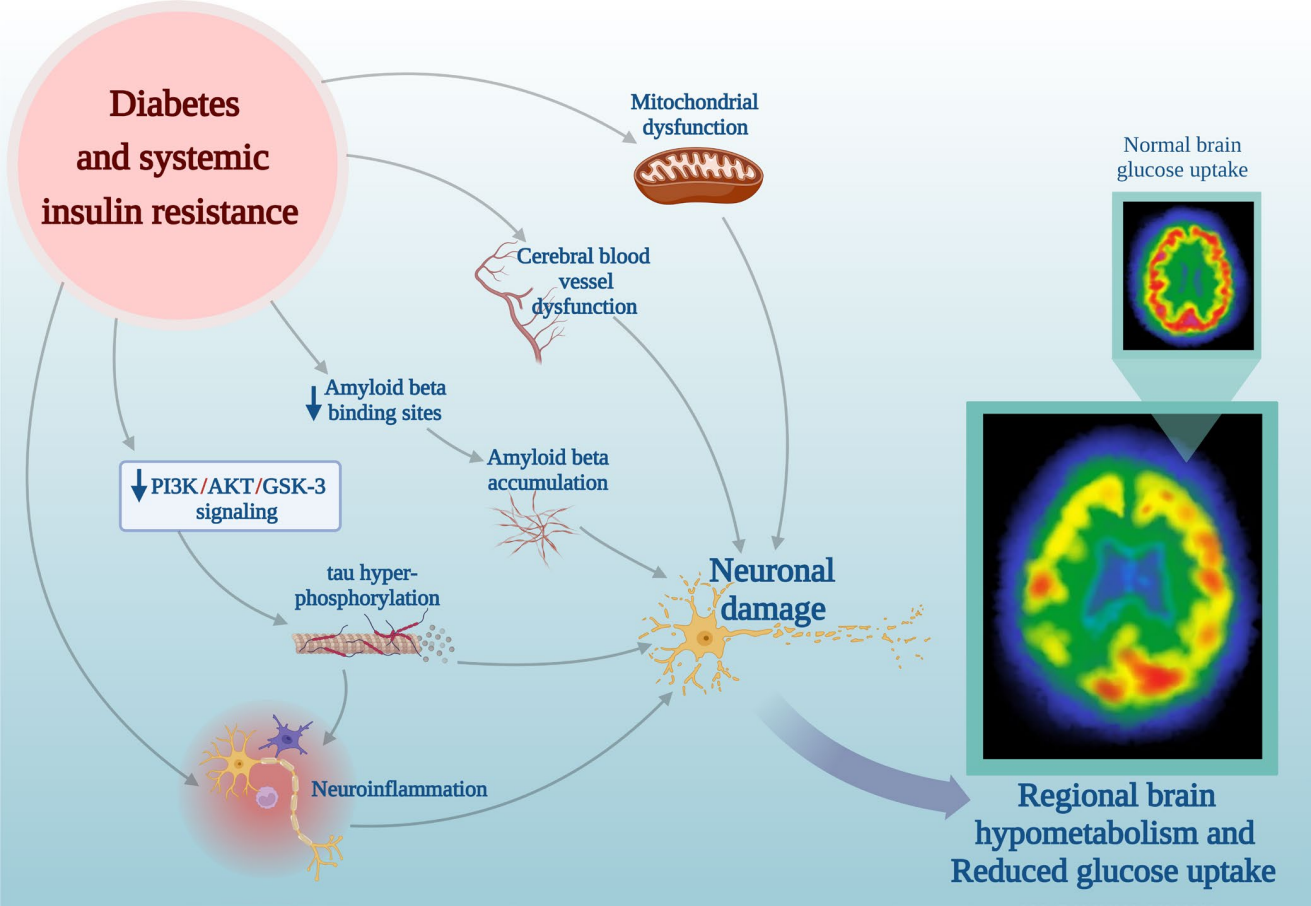
Potential links between insulin resistance and neuronal damage, leading to cerebral hypometabolism.

However, the pattern of lower cerebral FDG uptake due to higher blood glucose levels may be explained by three potential mechanisms: First, there are regional differences in the magnitude of competition between glucose and 18F‐FDG, which relate to the altered expression of glucose transporters and hexokinase (Ishibashi et al. 2019). Both diabetes and AD are associated with changes in the expression of glucose transporters (Koepsell 2020), and the resultant energy deficiency may contribute to the pathogenesis of AD. For instance, animal studies suggest that diabetes is associated with changes in the regulation of glucose transporter 4 (GLUT4) in the hippocampus (van der Graaf et al. 2004; Winocur et al. 2005; Patel et al. 2016). Second, alterations in the pattern of regional brain activity due to IR and prolonged hyperglycemia can lead to neuronal damage and a subsequent decline in brain activity (Figure 2) (Heurling et al. 2020; Rebelos et al. 2021); one mechanism is that higher insulin levels can bind to insulin‐degrading enzyme, which also has an active site for Aβ peptides, thereby down‐regulating the breakdown of Aβ plaques by this enzyme (Tian et al. 2023). Also, IR leads to decreased PI3K/AKT/GSK‐3 activity and downregulated Aβ oligomer binding sites in the synapse, which causes tau protein hyperphosphorylation and accumulation of Aβ, respectively, and further leads to the development of AD (Smolina et al. 2015; Yang et al. 2018); in addition, prolonged hyperglycemia and IR also lead to mitochondrial dysfunction, cerebral blood vessel dysfunction, and neuroinflammation (Singh et al. 2022). Third, the alteration observed with the pattern of FDG uptake due to higher plasma glucose levels could possibly be caused by a protective mechanism for more sensitive regions, by altered cerebral blood flow, intended to maintain normal glucose tissue utilization in these regions (Ishibashi et al. 2018; Heurling et al. 2020).

Another point to keep in mind in the interpretation of the results of FDG PET is that both the neuronal cells and glial cells, activated in response to the buildup of amyloid, use it, and thus, it cannot be concluded whether these observations are reflective of neurodegeneration, as they can be due to glial activation in response to amyloid accumulation. More studies are needed to consider the amyloid burden and neuroinflammation associated with IR and their potential effects on FDG uptake.

As FDG uptake is affected by plasma glucose levels, it is essential to precisely measure and monitor plasma glucose levels before the FDG‐PET scan and implement further normalization of blood glucose (Heurling et al. 2020). The current strategy is to operate brain FDG‐PET only in patients with blood glucose levels < 160 mg/dL (Varrone et al. 2009). However, this systematic review reveals a decline in brain SUV even in mild hyperglycemia (glycemic range of < 130 mg/dL) (Sprinz et al. 2018). Therefore, it is necessary to seek better normalization methods for the brain FDG‐PET in future studies.

Despite its strengths, there are some limitations to this systematic review. Approximately 22% of the studies included in this review were sourced from similar or partially overlapping samples of participants from the ADNI database, which might have potentially impacted certain findings and caused an overestimation of findings only from this database (Willette, Modanlo et al. 2015; Li et al. 2016; Apostolova et al. 2018; Sundermann et al. 2021; Rajendrakumar et al. 2024). In addition, despite our attempts to incorporate both significant and non‐significant findings, it is common to observe a preference for publishing papers with significant results, a phenomenon known as publication bias. Furthermore, it should be noted that the studies discussed in this review may have certain intrinsic limitations that warrant attention in future research. First, most of the literature did not assess the potential for cerebral hypometabolism‐related future cognitive decline, as only a few of the included studies had prospective follow‐up. The methodologies for FDG PET acquisition and analyses, like attenuation correction, partial volume correction, and the reference region, are possible sources of heterogeneity that may partly explain the variation in the results. Some studies reported their methodologies in detail and repeated their analyses using different reference regions or with and without partial volume correction, which yielded different results. However, among the studies reporting these details, there was no particular pattern of positive or negative results. Additionally, several possible confounding factors, such as the presence or absence of AD and MCI, the presence of APOE‐ε4 carriers, a positive family history of AD, the presence of comorbidities like cardiovascular diseases, depression profiles of participants, patients' age and sex, BMI, and medications like hormonal therapy, might have influenced FDG‐PET findings.

## 5 Conclusion

In conclusion, diabetes and IR are associated with cerebral glucose hypometabolism in similar regions compared to AD. This suggests that even in cognitively normal individuals, IR can contribute to altered glucose metabolism, which is one of the key preclinical AD changes. Further longitudinal studies are required to fully understand the relationship between cerebral hypometabolism and glycemic impairment, as well as the progression to cognitive decline and AD. Moreover, studies that investigate the association between impaired glycemic status and AD pathology, including beta‐amyloid and tau depositions, can be beneficial to understanding the mechanisms of cerebral hypometabolism associated with IR.

## Ethics Statement

The authors have nothing to report.

## Consent

The authors have nothing to report.

## Conflicts of Interest

The authors declare no conflicts of interest.

## Supporting information


**Table S1.** Newcastle–Ottawa Scale (NOS) risk of bias assessment for cross‐sectional studies.

**Table S2.** Newcastle–Ottawa Scale (NOS) risk of bias assessment for case–control studies.

**Table S3.** Newcastle–Ottawa Scale (NOS) risk of bias assessment for cohort studies.

**Data S1.** Supporting Information.

## Data Availability

Data sharing is not applicable to this article as no new data were created or analyzed in this study.
