# Supplementary material for: Relationships Between Brain Glucose Metabolism Patterns and Impaired Glycemic Status: A Systematic Review of FDG‐PET Studies With a Focus on Alzheimer's Disease
Source: Hum Brain Mapp. 2025 Mar 3;46(4):e70180. doi: 10.1002/hbm.70180 (PMC11876560; doi:10.1002/hbm.70180)
Supplement: Supplementary file 1 — Table S1. Newcastle–Ottawa Scale (NOS) risk of bias assessment for cross‐sectional studies. [file HBM-46-e70180-s001.pdf]

Supplementary Table 1. Newcastle-Ottawa Scale (NOS) risk of bias assessment for cross-sectional studies

Cross-sectional NOS	Willette2015	Burns2013	Apostolova2018	Ishibashi2017	Li2016	Waqas2019	Palix2022	Chen2022	Ishibashi2016	Ennis2021	Rajendrakumar202	Karayiannis2024	Viglianti2019
**Selection** (5*)													
Representativeness of cases (*)	/	*	*	/	*	*	*	*	/	/	*	*	*
Sample size adequacy (*)	/	/	*	/	*	/	/	/	/	/	/	/	/
Response rate > 95% (*)	*	*	*	*	*	*	*	*	*	*	*	*	*
Validity of screening tool (**)	**	**	**	**	**	**	**	**	**	**	**	**	**
**Comparability** (1*)													
Controlled for potential confounders (*)	*	*	*	*	*	*	*	*	*	*	*	*	*
**Outcome** (3*)													
Independent blind assessment or record link (**)	**	**	**	**	**	**	**	**	**	**	**	**	**
Apropriate statistical test (*)	*	*	*	*	*	*	*	*	*	*	*	*	*
Total quality score (out of 9)	7	8	9	7	9	8	8	8	7	7	8	8	8
