# Supplementary material for: Relationships Between Brain Glucose Metabolism Patterns and Impaired Glycemic Status: A Systematic Review of FDG‐PET Studies With a Focus on Alzheimer's Disease
Source: Hum Brain Mapp. 2025 Mar 3;46(4):e70180. doi: 10.1002/hbm.70180 (PMC11876560; doi:10.1002/hbm.70180)
Supplement: Supplementary file 2 — Table S2. Newcastle–Ottawa Scale (NOS) risk of bias assessment for case–control studies. [file HBM-46-e70180-s003.pdf]

Supplementary Table 2. Newcastle-Ottawa Scale (NOS) risk of bias assessment for case-control studies

Case-control NOS	Baker2011	Ishibashi2015	Képes2021	Roberts2014	Garcia-Casares2014	Castellano2015
**Selection (4*)**						
Case defenition (*)	*	*	*	*	*	*
Representativeness of cases (*)	*	/	*	*	*	/
Selection of controls (*)	/	/	*	*	*	/
Control defenition(*)	*	*	*	*	*	*
**Comparability** (2*)						
Control for the most important factor (*)	*	*	*	*	*	*
Matched for potential confounders (*)	*	/	*	*	*	*
**Exposure** (3*)						
Ascertainment of exposure (*)	*	*	*	*	*	*
Same method of ascertainment for cases and controls (*)	*	*	*	*	*	*
Non-Response rate (*)	*	*	*	*	*	*
Total quality score (out of 9)	8	6	9	9	9	7
