# Supplementary material for: Relationships Between Brain Glucose Metabolism Patterns and Impaired Glycemic Status: A Systematic Review of FDG‐PET Studies With a Focus on Alzheimer's Disease
Source: Hum Brain Mapp. 2025 Mar 3;46(4):e70180. doi: 10.1002/hbm.70180 (PMC11876560; doi:10.1002/hbm.70180)
Supplement: Supplementary file 3 — Table S3. Newcastle–Ottawa Scale (NOS) risk of bias assessment for cohort studies. [file HBM-46-e70180-s004.pdf]

Supplementary Table 3. Newcastle-Ottawa Scale (NOS) risk of bias assessment for cohort studies

Cohort-NOS	Burns2018	Sundermann2021	Rasgon2014	Willette2015
**Selection** (4*)				
Representation of exposed group (*)	*	*	/	/
Control (non-exposed) selection (*)	*	*	*	*
Ascertainment of exposure (*)	*	*	*	*
Outcome of interest was not present at start (*)	*	*	/	/
**Comparability** (2*)				
Control for the most important factor (*)	/	*	*	*
Matched for confounder factors or age (*)	*	/	*	*
**Outcome** (3*)				
Conformation of outcome by reference (*)	*	*	*	*
Long enough follow-up (*)	*	*	/	*
Adequacy of follow-up (*)	*	*	*	*
Total quality score (out of 9)	8	8	6	7
