# Supplementary material for: Relationships Between Brain Glucose Metabolism Patterns and Impaired Glycemic Status: A Systematic Review of FDG‐PET Studies With a Focus on Alzheimer's Disease
Source: Hum Brain Mapp. 2025 Mar 3;46(4):e70180. doi: 10.1002/hbm.70180 (PMC11876560; doi:10.1002/hbm.70180)
Supplement: Supplementary file 4 — Data S1. Supporting Information. [file HBM-46-e70180-s002.docx]

| FDG-PET scan brain findings | In CN adults, higher glycemic levels (FBS) were associated with atrophy and hypometabolism in AD-related regions including precuneus, cuneus, posterior cingulate cortex, angular cortex, and medial temporal gyrus. | HOMA-IR was not directly associated with the level of ^18^F-FDG uptake in brain. Lower glucose-stimulated insulin secretion was related to higher cerebral ^18^F-FDG uptake (in all ROIs except hippocampus) in middle adulthood (but not older adulthood) and higher RAVLT memory scores in a middle aged and older adults | Insulin resistance was associated with lower cerebral ^18^F-FDG uptake in bilateral middle temporal gyrus, bilateral middle frontal gyrus, right precentral gyrus, right inferior frontal gyrus, right cuneiform lobe and bilateral cerebellar regions. | Higher HOMA-IR was associated with lower regional cerebral ^18^F-FDG uptake in frontal, lateral parietal, lateral temporal, and especially left medial temporal lobe (independent of APOE-ε4 or family history status). Adjusted glucose metabolism in left MTL was associated with the immediate memory factor, the verbal learning and memory factor score,  and weakly with the Speed and flexibility factor score, but not with the working memory factor score. | DM was associated with lower median brain volume of 28% and lower median brain SUV max of 42%, compared with the non-diabetic |  |
| --- | --- | --- | --- | --- | --- | --- |
| iMAGE PROCESSING ANALYSIS method | Voxel-wise regression analysis of SUVRs from attenuation-corrected images controlling for age, sex, years of education, BMI, APOE-ε4 status | Attenuation-corrected image SUVRs, bilateral ROIs (the frontal  cortex, lateral  temporal  cortex, supramarginal  and angular gyri, precuneus, posterior cingulate  and hippocampus) | Voxel-wise and whole-brain SUV _max_ difference based on attenuation-corrected images in insulin-resistant and control groups followed by superimposition on MRI template | Whole-brain and voxel-wise analysis on attenuation-corrected images with age, sex, and BMI as covariates and APOE-ε4/family history as interaction terms | Whole-brain SUV _max_ and FBS*SUV _max,_ and automatic ROI |  |
| FASTING DURATION | 6 hours | - | 8 hours | 4 hours | 6 hours |  |
| SCANNING DURATION | 10 min at 50 min after FDG injection | 30 min at 30 min after FDG injection | Scanning performed 45-60 min after FDG injection | 30 min at 45 min after FDG injection | Scanning performed 60 min after FDG injection |  |
| route AND DOSE of FDG injection, | IV injection of 5 mCi ^18^F-FDG | IV injection of 185 MBq 18F-FDG | IV injection of 5.55 MBq/kg ^18^F-FDG | IV injection of 5.0 ± 0.5 mCi ^18^F-FDG | IV injection of 3.5 MBq/kg ^18^F-FDG |  |
| HBA1C (MEAN%), HOMA-IR (MEAN ± SD) | - | • HOMA-IR**:** 0.86 ± 0.34 | •HbA1C: 5.63 ± 0.83  •HOMA-IR: 1.96 ± 1.59 | • HOMA-IR: 2.2 ±1.9 | - |  |
| BS levels mg/dl (mean ± SD) | FBS: 1) 95.4 ± 12.6  2) 99 ± 9 | FBS: 93.52 ± 6.16 | FBS: 105.12 ± 21.6 | FBS: 94.6 ± 10.0 | FBS (in men): 102.6 ± 18  FBS (in women): 100.8 ± 23.4  *Only participants with FBS <10 mmol/L were included. |  |
| Cognitive status/MMSE score (mean ±SD) | CN: 105  AD: 18  MCI: 27 (based on the Petersen criteria) | CN (UDS assessment based on cognitive testes including Trail Making Test Part B, animal and vegetable fluency, Rey Auditory Verbal Learning Test) | CN (MMSE > 27) | CN (based on interview for working memory, immediate memory, speed and flexibility, and verbal learning, and not meeting NINCDS-ADRDA criteria for AD diagnosis) | CN (exclusion of those with cognitive impairment or dementia) |  |
| Positive FAMILY HISTORY OF AD OR APOE-ε4 CARRIERS (NUMBER) | • ApoE4-ε4 carriers: 1) 34  2)28 | • APOE-ε4 carriers: 22 | - | • Positive family history: 103  • APOE-ε4 carriers: 61 | - |  |
| BMI (mean ± SD) | 1) 23.6 ±2.9  2) 24.4 ± 3 | - | 24.82 ± 2.92 | 28.2 ± 5.3 | 27 ± 5.3 |  |
| Glycemic status (DM or non-DM or pre-diabetic) | Non-DM: 150 | Prediabetic: 22 | DM: 25  Non-DM: 164 (propensity-score matched) | DM: 7  Non-DM: 143 | DM: 33 (14 male/ 19 female) |  |
| Education years (mean ± SD) | 1) 13 ± 3.3  2) 11.6 ± 3. | 16.80 ± 2.11 | - | - | - |  |
| Age years (mean ± SD) | 1. 49.2 ± 20.1   2) 70.8 ± 8.6 | 63.4 ± 4.9 | 48.68 ± 8.96 | 60.67 ± 5.82 | 66 ± 3.3 (male: 66 ±6/ female: 66 ±7)  *Only participants with age 60-70 years were included |  |
| Number of participants (male/female) | 150 (86 male/64 female) | 69 (29 male/40 female) | 189 (129 male/60 female) | 150 (42 male/ 108 female) | 119 (56 male/ 63 female) |  |
| Groups | 1) CN (n=105)  2) Cognitive impairment with amyloid beta accumulation assessed by florbetapir PET (AD=18, MCI=27) | - | - | - | - |  |
| study design | Cross-Sectional | Cross-Sectional | Cross-Sectional | Cross-Sectional | Cross-Sectional |  |
| year | 2022 | 2021 | 2022 | 2015 | 2019 |  |
| First Author | Palix et al. | Ennis et al. | Chen et al. | Willette et al. | Waqas et al. |  |

| FDG-PET scan brain findings | Higher HOMA-IR was associated with lower CMRglu in frontal, temporal-parietal, and cingulate regions (independent of age, 2-h OGTT glucose, or APOE-ε4).  Better recall was associated with greater CMRglu for right frontal and posterior cingulate cortices and for left frontal, temporal, and parietal cortices. | Higher fasting serum glucose levels were significantly correlated with lower regional CMRglu in right temporal areas and bilaterally in precuneus, posterior cingulate, parietal, prefrontal, and occipital brain regions (independent of APOE-ε4 status) | IFG and AD groups showed lower regional cerebral ^18^F-FDG uptake in the precuneus compared to normal control group. AD group showed lower ^18^F-FDG uptake in precuneus cortex compared to IFG group. | DM individuals had lower cerebral ^18^F-FDG uptake in precuneus, left posterior orbital gyrus, right calcarine cortex and right orbital part of inferior frontal gyrus and superior frontal gyrus in voxel-wise analysis, but after correction for pre-PET glucose levels, only right superior frontal gyrus showed hypometabolism. Pre-PET glucose was associated with lower metabolism in precuneus cortex. Regions showing glucose sensitive hypometabolism in diabetic group: Precuneus/posterior cingulate gyrus, left posterior orbital gyrus, right calcarine cortex, right orbital part of the inferior frontal gyrus  Glucose sensitive reduced metabolism in non-diabetic obese group: right Rolandic operculum | |
| --- | --- | --- | --- | --- | --- |
| iMAGE PROCESSING ANALYSIS method | Voxel-wise regression analysis of CMRglu on attenuation-corrected images | Voxel-wise regression analysis of CMRglu maps followed by superimposition of a standardized MRI volume | Whole-brain voxel-wise between-group and regression analysis of SUVRs from attenuation-corrected images followed by ROI analysis (visual cortex used as the reference region) | Regional (based on NeuroQ software) and voxel-wise analysis on attenuation-corrected images followed by superimposition on T1 MRI |  |
| FASTING DURATION | An overnight fast | 4 hours | - | - |  |
| SCANNING DURATION | 30 min at 40 min after FDG injection | 60 min | Scanning performed 45-51 min after FDG injection | Scanning performed 45 min after FDG injection |  |
| route AND DOSE of FDG injection | IV injection of 5 mCi ^18^F-FDG | IV injection of 5-8 mCi ^18^F-FDG | IV injection of 150 MBq ^18^F-FDG | IV injection of 3.5 MBq/kg ^18^F-FDG |  |
| HBA1C (MEAN%), HOMA-IR (MEAN ± SD) | - | - | - | •HbA1C:  1) 7.59 ± 1.27  2) 5.49 ± 0.33 |  |
| BS levels mg/dl (mean ± SD) | •FBS: 1) 107.14 ± 12.3  2) 100.95 ± 6.1  • 2h-OGTT: 1) 187.78 ± 48.3   1. 110.66 ± 14.6 | FBS: 1a) 96.7 ± 10  1b) 98.1 ± 10.9  2) 96.1 ± 9.2 | FBS for impaired fasting glucose group: 103.9 ± 2.21  FBS for normal FBS group: 89.0 ± 5. | FBS: 1) 152.1 ± 80.64  2) 97.2 ± 13.5 |  |
| Cognitive status (MMSE score mean ±SD) | CN: 6  Subtle cognitive impairment: 23 (based on Petersen criteria) | •MMSE scores:  1a) 29.5 ± 1  1b) 29.6 ± 1  2) 29.6 ± 0.9 | CN (MMSE score > 27): 51  AD: 15 | CN (no history of mental or neurologic disorder) |  |
| Positive FAMILY HISTORY OF AD OR APOE-ε4 CARRIERS (NUMBER) | - | •Positive family history:  124  • APOE-ε4 carriers: 63 | - | - |  |
| BMI (mean ± SD) | 1. 27 ± 2.9 2. 28.5 ± 3.4 | - | - | 1) 33.87  2) 38. 35 |  |
| Glycemic status (DM or non-DM or pre-diabetic) | Pre-diabetic: 11  DM: 12  Non-DM: 6 | Non-DM | Pre-diabetic: 20  Non-DM: 46 | DM: 51  Non-DM: 45 |  |
| Education years (mean ± SD) | - | 1a) 15.7 ± 2.1  1b) 16.3 ± 2.2  2) 15.9 ± 2.1 | - | - |  |
| Age years (mean ± SD) | 1. 74.39 ± 7.1 2. 74.33 ± 6.3 | 1a) 63.1 ± 6.3  1b) 64.3 ± 6  2) 64.2 ± 6.2 | 1) 68.1 ± 6.2  2) 72.2 ± 4.6   1. 69.7 ± 5.9 | 1) 50.6 ± 8.0  2) 52+ 9.6 |  |
| Number of participants (male/female) | 29 | 124 | 66 (8 male/ 58 female) | 96 |  |
| Groups | 1. DM and pre-diabetic (n=23) 2. Non-DM (n=6) | 1) ApoE4 carriers: 1a) Homozygotes (n=23)  1b) ApoE4 carriers- Heterozygote (n=40)  2) ApoE4 non carriers (n=61) | 1) CN with normal FBS (n=31)  2) CN with IFG (n=20)  3) AD (n=15) | 1. DM (n=51) 2. Non-DM obese (n=45) |  |
| study design | Case Control | Cross-Sectional | Case Control | Case Control |  |
| year | 2011 | 2013 | 2015 | 2021 |  |
| First Author | Baker et al. | Burns et al. | Ishibashi et al. | Képes et al. |  |

| FDG-PET scan brain findings | BS levels (59–149 mg/dl) were negatively associated with the regional cerebral ^18^F-FDG uptake in the occipital cortex and precuneus but sparing the posterior cingulate, independent of amyloid-β and APOE-ε4 status.  Blood glucose-associated variability of FDG uptake had no relevant impact on the prediction of AD progression in MCI. | There was a negative correlation of plasma glucose levels with ^18^F-FDG uptake in the precuneus (4-5 % reduction by 20 mg/dL increase in glucose) and lateral parietotemporal regions, but no correlation between glucose metabolism and plasma insulin levels or HOMA-IR. | DM group (irrespective of cognitive status) showed lower regional cerebral ^18^F-FDG uptake in the AD meta-ROI in APOE-ε4 carriers. In CN non-diabetics, the odds of hypometabolism in the AD signature meta-ROI increased with each 1% increase in HBA1c after adjustment for age, sex, APOE-ε4 status and education. With partial volume correction, glucose-adjusted models did not show significance. | There was a negative correlation of CMRglu with HOMA-IR in the right superior frontal, left and right middle frontal, left supramarginal, left middle temporal cortices as well as the amygdala and the hippocampus; the PCOS group had higher IR and consequently 9-14% lower regional cerebral glucose metabolism. |
| --- | --- | --- | --- | --- |
| iMAGE PROCESSING ANALYSIS method | Voxel-wise regression analysis followed by ROI analysis controlling for age, gender, cognitive performance, ApoE-ε4 and amyloid status | •Voxel-wise regression analysis followed by ROI analysis of attenuation-corrected images (without partial volume correction) | AD meta-ROI (the average bilateral angular  gyri, posterior cingulate/precuneus, and inferior temporal cortical ROIs from both  hemispheres, normalized to pons uptake) on attenuation-corrected images with and without partial volume correction. | ROI analysis of CMRglu based on MRI regions after partial volume correction |
| FASTING DURATION | 4 hours | 5 hours | - | 6-7 hours |
| SCANNING DURATION | 30 min at 30-60 min post-injection | Scanning performed at 10 min and 40 min post-injection | 30-38 min | 60 min |
| route AND DOSE of FDG injection | IV injection of 5.0 ± 0.5 mCi ^18^F-FDG | IV injection of 4 mCi ^18^F-FDG | - | IV injection of 5.6 ± 0.6 mCi ^18^F-FDG |
| HBA1C (MEAN%), HOMA-IR (MEAN ± SD) | - | • HOMA-IR: 1 ± 0.5 | - | •HbA1C: 1) 5.1 ± 0.2  2) 5.2 ± 0.2  • HOMA-IR: 1) 0.7 ± 0.3  2) 0.5 ± 0.3 |
| BS levels mg/dl (mean ± SD) | CN: 98.4 ± 15.8  MCI: 101.2±16.9 | FBS: 102.1 ± 8.1 | FBS: 1) 114.5 (median)  2) 97 (median) | FBS: 1) 81 ± 5.4  2) 73.8 ± 7.2 |
| Cognitive status (MMSE score mean ±SD) | CN: 87  MCI: 323  (cognitively stable for more than 36 months) | CN (MMSE scores > 27) | CN: 587  MCI: 162  (Clinical Dementia Rating Scale (CDR) and Functional Activities Questionnaire (FAQ) plus age-adjusted cognitive domain testing: MCI was defined as impairment in one or more domains) | CN (based on cognitive impairment was defined as score more than 1.65 standard deviations below the composite score normative values for age and education) |
| Positive FAMILY HISTORY OF AD OR APOE-ε4 CARRIERS (NUMBER) | • APOE-ε4 carriers (among CN participants): 24 | - | • APOE-ε4 carriers:  1) 40  2) 160 | - |
| BMI (mean ± SD) | - |  | - | 1) 24.5 ± 2.4  2) 23.6 ± 3.0 |
| Glycemic status (DM or non-DM or pre-diabetic) | - | Non-DM | DM: 154  Non-DM: 595 | Non-DM |
| Education years (mean ± SD) | - | - | 1) 13 (median)  2) 14 (median) | 1) 15 ± 1  2) 16 ± 1 |
| Age years (mean ± SD) | CN: 74.2± 5.3  MCI: 71.1±7.1 | 75.7 ± 6.4 | 79.0 (median) | 1) 24.6 ± 5.9  2) 24.0 ± 3.3 |
| Number of participants (male/female) | 410 (224 male/ 186 female) | 59 (10 male/49 female) | 749 (423 male/326 female) | 18 (female) |
| Groups | A cohort of CN: 87 (including 44 Amyloid negative, 24 amyloid-positive, and 19 without amyloid marker) and  MCI: 323 | - | 1) DM (n=154)  2) Non-DM (n=595) | 1) PCO patients (n=7)  2) Healthy control (n=11) |
| study design | Cross-Sectional | Cross-Sectional | Case Control | Case Control |
| year | 2018 | 2017 | 2014 | 2015 |
| First Author | Apostolova et al. | Ishibashi et al. | Roberts et al. | Castellano et al. |

| FDG-PET scan brain findings | Higher blood glucose levels were associated with lower cerebral ^18^F-FDG uptake in the precuneus/posterior cingulate, lateral parietal cortex, and frontal cortex. | Participants with MCI + T2DM showed lower whole-brain, frontal lobe, sensory motor cortex, and striatum ^18^F-FDG SUVR relative to MCI without T2DM. T2DM showed no effects within either CN or AD groups. | There was a negative inverse relationship between ^18^F-FDG uptake in regions such as post-central and precentral gyrus, hippocampus, amygdala, angular gyrus, medial temporal, inferior temporal, parietal lobe, and hyperglycemia. This significant association was only present when whole-brain was used as the reference region (not cerebellum, or pons). | Diabetes group had lower regional cerebral ^18^F-FDG uptake in the orbital and left prefrontal cortex, premotor, temporal lobe (middle and inferior gyrus, parahippocampus and uncus), and cerebellum regions. Also, diabetes group had lower recall scores correlating with frontal, temporal, and cingulate hypometabolism. |
| --- | --- | --- | --- | --- |
| iMAGE PROCESSING ANALYSIS method | Voxel-wise followed by ROI (precuneus/posterior cingulate, lateral parietal, and frontal cortex) analysis of attenuation-corrected images | Mean SUVR values of frontal, parietal, temporal, occipital, and limbic lobe and whole brain with pons as the reference region | ROI (manually drawn) analysis on attenuation corrected images using SUV based on body weight (SUVkg). The z-scores derived from each region SUVR with whole-brain, pons, or cerebellum as the reference region was used. | Voxel-wise analysis on attenuation-corrected images after normalization of global counts to 50mg/mL/min and partial volume correction |
| FASTING DURATION | 5 hours | 4 hours | - | 6 hours |
| SCANNING DURATION | 6 min at 45 min post-injection | 30 min at 30-60 min post-injection | - | 20 min at 40 min post-injection |
| route AND DOSE of FDG injection | IV injection of 4 mCi ^18^F-FDG | IV injection of 5.0 ± 0.5 mCi ^18^F-FDG | IV injection of an average of 12.8 ± 0.16 mCi ^18^F-FDG | IV injection of 370 MBq ^18^F-FDG |
| HBA1C (MEAN%), HOMA-IR (MEAN ± SD) | - | - | - | •HbA1C: 1) 6.67 ± 0.76  2) 5.32 ± 0.  07 |
| BS levels mg/dl (mean ± SD) | Ranges: 80-226 | FBS in T2DM patients using antidiabetic medications: 118.74 ± 35.69  FBS in T2DM patients not using antidiabetic medications: 106.79 ± 18.70 | FBS in DM patients: 103 ± 6.24  FBS in non-DM patients: 149.5 ± 6.87 | - |
| Cognitive status (MMSE score mean ±SD) | • MMSE scores: > 27 | CN: 380  MCI: 791  AD: 107 (based on CDR, MMSE, and cognitive scores) | CN (excluded if the PET/CT was done for neurodegenerative disease evaluation, history of or suspected dementia, cerebral vascular accident) | •MMSE score ≥ 26: 1) 28.8 ± 1.3  2) 29.7 ± 0.7 |
| POSITIVE FAMILY HISTORY OF AD OR APOE-ε4 CARRIERS (NUMBER) | - | • APOE-ε4 carriers: 772 | - | • APOE-ε4 carriers: 1) 9  2) 5 |
| BMI (mean ± SD) | - | - | DM: 28.8 ± 1.33  Non-DM: 25.4 ± 1.03 | 1) 28.6 ± 4.1  2) 26 ± 3.2 |
| Glycemic status (DM or non-DM or pre-diabetic) | DM: 4 | DM: 112  Non-DM: 1166 | DM: 29  Non-DM: 24 | DM: 25  Non-DM: 25 |
| Study duration | 7.75 ± 2.27 | - | - |  |
| Education years (mean ± SD) | - | 1. CN: 16.16 ± 0.14  2. CN + T2DM: 16.42 ± 0.51  3. MCI: 15.96 ± 0.09  4. MCI + T2DM: 15.30 ± 0.28  5. AD: 14.84 ± 0.20  6. AD + T2DM: 13.21  ± 0.75 | - | 1) 18.9 ± 3.6  2) 18.9 ± 4 |
| Age years (mean ± SD) | Age ranges at the beginning of the study: 67-69 | 1. CN: 74.27 ± 0.36  2. CN+ T2DM: 74.81 ± 1.30  3. MCI: 72.81 ± 0.24  4. MCI + T2DM: 71.84 ± 0.72  5. AD: 74 ± 0.52  6. AD + T2DM: 78 ± 1.93 | DM patients: 65.7 ± 2.79  Non-DM patients: 60.8 ± 4.31 | 1) 60 ± 4.1  2) 57.8 ± 5.1 |
| Number of participants (male/female) | 4 (1 male/ 3 female) | 1278 (925 male/ 741 female) | 53 (53 male) | 50 (31 male/19 female) |
| Groups | - | 1. CN (n=349)  2. CN + T2DM (n=31)  3. MCI (n=719)  4. MCI + T2DM (n=72)  5. AD (n=98)  6. AD + T2DM (n=9) | 1. DM (n=29)  2. Non-DM (n=24) | 1. DM (n=25)  2. Non-DM (n=25) |
| study design | Case series | Cross-Sectional | Cross-Sectional | Case Control |
| year | 2016 | 2016 | 2019 | 2014 |
| First Author | Ishibashi et al. | Li et al. | Viglianti et al. | García – casares et al. |

| FDG-PET scan brain findings | T2DM was associated with lower whole brain, frontal, parietal, temporal and  Occipital ^18^F-FDG uptake and poorer attention. | Higher blood glucose and HDL-C levels were associated with lower cerebral 18F-FDG uptake. Being an APOE4 allele carrier was also a predictor of brain hypometabolism. |  |  |
| --- | --- | --- | --- | --- |
| iMAGE PROCESSING ANALYSIS method | Voxel-wise followed by ROI (frontal lobe,  parietal lobe, temporal lobe, limbic lobe, and occipital  lobe) analysis of attenuation-corrected images | Voxel-wise whole brain image analysis |  |  |
| FASTING DURATION | - | - |  |  |
| SCANNING DURATION | - | - |  |  |
| route AND DOSE of FDG injection | IV injection of 250 MBq of ^18^FDG | - |  |  |
| HBA1C (MEAN%), HOMA-IR (MEAN ± SD) | •HbA1C: 1) 7.1 ± 1.4  2) 5.6 ± 0.3  • HOMA-IR: 1) 1.7 ± 1.2  2) 1.1 ± 1.4 | •HbA1C: 5.42 |  |  |
| BS levels mg/dl (mean ± SD) | FBS in DM patients: 142.2 ± 46.8  FBS in non-DM patients: 97.2 ± 9.0 | FBS: 97.56 ± 15.66 |  |  |
| Cognitive status (MMSE score mean ±SD) | CN, Subtle reductions in attentional function in T2DM patients based on comprehensive set of cognitive battaeries | CN: 260  AD: 76 (based on CDR, MMSE, and cognitive scores) |  |  |
| POSITIVE FAMILY HISTORY OF AD OR APOE-ε4 CARRIERS (NUMBER) | - | • APOE-ε4 carriers: 163 |  |  |
| BMI (mean ± SD) | DM: 34.4 ± 5.9  Non-DM: 29.8 ± 5.7 | - |  |  |
| Glycemic status (DM or non-DM or pre-diabetic) | DM: 23  Non-DM: 23 | Non-DM: 336 |  |  |
| Education years (mean ± SD) | - | 15.66 ± 2.96 |  |  |
| Age years (mean ± SD) | 63.7 ± 6.1 | 75.43 ± 6.69 |  |  |
| Number of participants (male/female) | 46 (19 male/ 27 female) | 336 (215 male/ 121 female) |  |  |
| Groups | 1) DM (n=23)  2) Non-DM (n=23) | - |  |  |
| study design | Cross-Sectional | Cross-Sectional |  |  |
| year | 2024 | 2024 |  |  |
| First Author | Karayiannis et al. | Rajendrakumar et al. |  |  |

**Abbreviations**: AD Alzheimer’s disease, BS blood sugar, CMRglu cerebral metabolic rate of glucose, CN cognitively normal, DM diabetes mellitus, FBS fasting blood sugar, HOMA-IR homeostatic model assessment of insulin resistance, IFG impaired fasting glucose, IR insulin resistance, IV intravenous, MCI mild cognitive impairment, MMSE mini-mental state examination, OGTT oral glucose tolerance test, ROI region of interest, SUV standard uptake value, SUVR standard uptake value ratio, T2DM type 2 diabetes

| FDG-PET scan brain findings | Longitudinal increases in serum glucose levels were associated with longitudinal regional cerebral ^18^F-FDG uptake decline in the vicinity of parietotemporal, precuneus/posterior cingulate, and prefrontal brain regions (independent of APOE-ε4 status) | Pre-diabetes was associated with lower regional cerebral ^18^F-FDG uptake in the Meta-ROI and higher cognitive decline especially in women. Also, predabetes was associated with more rapid longitudinal decline in glucose metabolism and changes in cognitive scores especially in the MCI women. | Higher HOMA-IR (>2.3) was associated with greater cerebral ^18^F-FDG uptake decline over 2-year follow up. Continued estrogen-based hormonal therapy protected cerebral glucose metabolism in low HOMA-IR compared with discontinued hormonal therapy with low HOMA-IR. | Higher HOMA-IR was associated with hypermetabolism in MCI-progressors to AD (n=39) in MTL and hippocampus and hypermetabolism in stable MCI group.  Higher HOMA-IR was associated with less ^18^F-FDG uptake in MTL, Lateral Parietal and Posteromedial, and vPFC, for AD participants, whereas associations were nonsignificant for cognitively normal or MCI participants. | |
| --- | --- | --- | --- | --- | --- |
| iMAGE PROCESSING ANALYSIS method | Voxel-wise analysis | Linear mixed models for comparing average SUVR in MetaROI (bilateral posterior cingulate gyrus,  bilateral angular gyrus, and middle/inferior temporal gyrus) | Voxel-wise analysis and ROI (left and right medial prefrontal gyrus) on attenuation-corrected images | •ROI (bilateral hippocampus, MTL, lateral  parietal, posteromedial (precuneus and posterior cingu-  late cortex), and vPFC. Also, whole cerebrum and postcentral gyrus were used as control ROIs) | |
| FASTING DURATION | 4 hours | 4 hours | 4-6 hours | 4 hours | |
| SCANNING DURATION | 60 min | 30 min at 30-60 min post-injection | Scanning performed at 40 min post-injection | 30 min at 30-60 min post-injection | |
| route AND DOSE of FDG injection | IV injection of 5–8 mCi ^18^F-FDG | IV injection of 5.0 ± 0.5 mCi ^18^F-FDG | IV injection of 370 MBq ^18^F-FDG | IVinjection of 5.0 ± 0.5 mCi ^18^F-FDG | |
| HBA1C (MEAN%), HOMA-IR (MEAN ± SD) | - | - | **-** | • HOMA-IR: cognitively normal: 0.87 ± 1.10  MCI: 0.71 ± 0.84  AD: 0.60 ± 0.40 | |
| Blood sugar levels mg/dl (mean ± SD) | Primary FBS: 91.0 ± 8.0,  Secondary FBS (over the course of the study ~ 4.4 years): 95.2 ± 8.6 | FBS: 1) 107.8 ± 6.7  2) 87.8 ± 11.1  3) 109.0 ± 6.5  4) 90.1 ± 8.5 | Baseline FBS: 91.46 ± 12.01  2-year follow up FBS: 95.47 ± 12.73 | FBS in CN participants: 104.6 ± 30.1  FBS in MCI participants: 101.1 ± 20.3  FBS in AD participants: 100.5 ± 20.8 | |
| Cognitive status (MMSE score mean ±SD) , MCI (number) | CN (MMSE score > 28)  (29.7 ± 0.7) | •MCI: 1) 58  2) 104  3) 98  4) 111    **•**MMSE score: 1) 28.3 ± 1.7  2) 28.5 ± 1.5  3) 28.1 ± 1.6  4) 28.2 ± 1.7 | CN (MMSE score > 24) | CN: 26  AD: 60  MCI: 187 |  |
| POSITIVE FAMILY HISTORY OF AD OR APOE-ε4 CARRIERS (number) | •Positive family history: 80  • APOE-ε4 carreiers: 38 | • APOE-ε4 carriers:  1) 75  2) 102  3) 87  4) 117 | **•**  APOE-ε4 carriers:  1) 4  2) 3  3) 4  4) 7 | • APOE-ε4 carriers:  CN participants: 3  MCI participants: 103  AD participants:43 | |
| BMI (mean ± SD) | 26.7±4.5 | 1) 27.4 ± 5.8  2) 26.5 ± 5.2  3) 27.5 ± 4.1  4) 26.8 ± 3.4 | - | Cognitively normal : 26.76 ± 3.48  MCI : 26.30 ± 3.93  AD : 26.08 ± 3.83 | |
| Glycemic status (diabetic or non-diabetic) | Non-DM | Prediabetic: 389  Non-DM: 522 | Non-DM | - | |
| Study duration | 4.4 ± 1.0 (SD) years | 1) 3.8 ± 2.4 years  2) 4.2 ± 2.4 years  3) 4.1 ± 2.6 years  4) 4.4 ± 2.9 years | 2 years | 3 years | |
| Education years (mean ± SD) | - 1. ± 2.0 | 1) 15.7 ± 2.7  2) 15.8 ± 2.6  3) 16.5 ± 2.7  4) 16.7 ± 2.6 | 1) 16.4 ± 2  2) 14.9 ± 1.6  3) 15.5 ± 2.5  4) 16.8 ± 1.9 | Cognitively normal: 15.34 ± 3.15  MCI: 15.77 ± 2.90  AD: 14.80 ± 3.45 | |
| Age years (mean ± SD) | 61.5 ± 5 | 1) 72.2 ± 6.7  2) 72.2 ± 7.3  3) 73.9 ± 7  4) 73.9 ± 6.9 | 1) 58.5 ± 4.5  2) 57.6 ± 4.8  3) 56 ± 1.8  4) 58.4 ± 6.6 | CN: 75.69 ± 5.68  MCI: 75.17 ± 7.29  AD: 75.25 ±7.26 | |
| Number of participants (male/female) | 80 (52 male/28 female) | 911 (492 male/ 419 female) | 42 (female) | 280 (95 male/185 female) | |
| Groups | 1. APOE ε4 allele carriers (n=38) 2. APOE ε4 allele noncarriers (n=42) | 1. Pre-diabetic women (n=167) 2. Non-DM women (n=252) 3. Pre-diabetic men (n=222) 4. Non-DM men (n=270) | 1) Continued hormonal therapy with low IR; HOMA-IR<2.3 (n=17)  2) Continued hormonal therapy with high IR; HOMA-IR>2.3 (n=9)  3) Discontinued hormonal therapy with low IR; HOMA-IR<2.3 (n=4)  4) Discontinued hormonal therapy with high IR; HOMA-IR>2.3 (n=12) | 1) CN participants (n=26)  2) Participants with MCI at baseline who remained stable by 24 months (n = 148)  3) Participants with MCI at baseline who progressed to AD by 24 months (n=39)  4) Participants with AD at baseline (n=60) | |
| study design | Prospective cohort | Prospective Cohort | Prospective Cohort | Cohort | |
| year | 2018 | 2021 | 2014 | 2015 | |
| First Author | Bums et al. | Sundermann et al. | Rasgon et al. | Willette et al. | |

**Abbreviations:** AD Alzheimer’s disease, BS blood sugar, CMRglu cerebral metabolic rate of glucose, CN cognitively normal, DM diabetes mellitus, FBS fasting blood sugar, HOMA-IR homeostatic model assessment of insulin resistance, IFG impaired fasting glucose, IR insulin resistance, IV intravenous, MCI mild cognitive impairment, MMSE mini-mental state examination, ROI region of interest, SPM statistical parametric mapping, SUV standard uptake value, SUVR standard uptake value ratio, T2DM type 2 diabetes, vPFC ventral prefrontal cortex
